# Large Reductions in Match Play Physical Performance Variables Across a Professional Football Season With Control for Situational and Contextual Variables

**DOI:** 10.3389/fspor.2020.570937

**Published:** 2020-10-15

**Authors:** Matthew Springham, Sean Williams, Mark Waldron, Darren Burgess, Robert Usher Newton

**Affiliations:** ^1^Faculty of Sport, Health and Applied Science, St Marys University, London, United Kingdom; ^2^School of Medical and Health Sciences, Edith Cowan University, Joondalup, WA, Australia; ^3^Department for Health, University of Bath, Bath, United Kingdom; ^4^College of Engineering, Swansea University, Swansea, United Kingdom; ^5^School of Science and Technology, University of New England, Armidale, NSW, Australia; ^6^Sport Science Department, Melbourne Football Club, Melbourne, VIC, Australia

**Keywords:** football, performance, load, demands, fatigue, match play, monitoring

## Abstract

This investigation examined match play physical performance across a professional football season using a multicamera computerized tracking system. A linear mixed-effects model, controlling for situational and contextual variables, identified decreases in team average total distance (TD): season quarter 1 (Q1) (11,047 m) > season quarter 2 (Q2) (10,473 m) (*P* = *0.002*; ES = *Small*), season quarter 3 (Q3) (10,449 m) (*P* < 0.001; ES = *Moderate*), and season quarter 4 (Q4) (10,385 m) (*P* < 0.001; ES = *Moderate*); work rate (WR): Q1 (115 m/min) > Q3 (108 m/min) (*P* < 0.001; ES = *Moderate*), Q4 (107 m/min) (*P* < 0.001; ES = *Moderate*); Q2 (109 m/min) > Q4 (107 m/min) (*P* = 0.003; ES = *Small*); high-speed running distance (HSR): Q1 (1,051 m) > Q2 (813 m) (*P* = 0.006; ES = *Small*); number of high-speed runs (NHSR): Q1 (87) > Q2 (65) (*P* < 0.001; ES = *Small*), Q3 (64) (*P* = 0.002; ES = *Small*); sprint distance (SD): Q1 (202 m) > Q4 (130 m) (*P* < 0.001; ES = *Moderate*), Q2 (179 m) > Q3 (165 m) (*P* = 0.035; ES = *Small*), Q4 (130 m) (*P* < 0.001; ES = *Moderate*) and number of sprints (NS): Q1 (20.4) > Q3 (10.2) (*P* < 0.001; ES = *Moderate*), Q4 (8.3) (*P* < 0.001; ES = *Large*); Q2 (14.9) > Q3 (10.2) (*P* < 0.001; ES = *Moderate*), Q4 (8.3) (*P* < 0.001; ES = *Large*). Within-position changes were observed for WR: Q1 (122 m/min) > Q4 (113 m/min) (*P* = 0.002; ES = *Large*) in central midfielders and for NS: Q1 > Q3 in wide defenders (21.7 vs. 10.8) (*P* = 0.044; ES = *Large*) and central midfielders (18.1 vs. 8.3) (*P* = 0.002; ES = *Large*); Q1 > Q4 in central defenders (13.1 vs. 5.3) (*P* = 0.014; ES = *Large*), wide defenders (21.6 vs. 7.1) (*P* < 0.001; ES = *Very Large*), central midfielders (18.1 vs. 8.5) (*P* = 0.005; ES = *Large*), and wide midfielders (20.8 vs. 12.2) (*P* = 0.012; ES = *Large*); Q2 > Q3 in central midfielders (16.9 vs. 8.3) (*P* = 0.002; ES = *Large*) and Q2 > Q4 in wide defenders (16.3 vs. 7.1) (*P* = 0.005; ES = *Very Large*), central midfielders (16.9 vs. 8.5) (*P* = 0.004; ES = *Large*), and wide midfielders (20.8 vs. 12.2) (*P* = 0.007; ES = *Large*). The match-play physical performance was reduced across the competitive season. The most notable reductions were observed in wide defenders, central midfielders, and wide midfielders in sprint performance indices.

## Introduction

The English Football League Championship (EC) is the second tier of professional Football Association (football) in England and is characterized by substantial physical demands. For example, compared to the other major European Leagues (the English Premier League, Scottish Premiership, Scottish Championship, Spanish La Liga, German Bundesliga, Italian Serie-A, Dutch Eredivisie, and French Ligue 1) (UEFA, 2016), the EC teams play the most competitive games, number of two-game weeks, and have the greatest fixture density (Springham et al., 2019). Match load demands in the EC are also noteworthy. For example, match play total (TD), high-speed running (HSR), and sprinting (SD) distances are greater in the EC than in the English Premier League (EPL) (Bradley et al., 2013).

Scientific literature indicates that 24–96 h of recovery is necessary to reestablish baseline levels in markers of recovery post football match play (Ascensao et al., 2008; Ispirlidis et al., 2008; Nedelec et al., 2012; Thorpe and Sunderland, 2012; Russell et al., 2015, 2016; Thorpe et al., 2015). However, EC teams play an average of ~1.3 games per week across a ~40-week season, including ~16 two-game weeks, between which only ~72 h of recovery is available (Springham et al., 2019). Consequently, it is evident that regularly selected players in the EC might frequently be required to train and play in a fatigued state during periods of high fixture density (Meeusen et al., 2013; Schwellnus et al., 2016; Soligard et al., 2016).

Research to date indicates that periods of high game density (for example, playing > 1 game/week) (Gregson et al., 2010; Morgans et al., 2014b; Hattersley et al., 2018) and high acute workload (Owen et al., 2016; Rowell et al., 2018; Springham et al., 2020) can increase fatigue and compromise match play physical performance in football players. For example, Hattersley et al. (2018) reported a 22% reduction in match play HSR during the second half of the second game during two-game weeks. Moreover, Gregson et al. (2010) reported a greater variability in match play HSR distance during periods of increased game density, and more recently, Springham et al. (2020) reported that high acute (7-day average) HSR and high metabolic loads have moderate compromising effects on subsequent high-intensity match performance. However, there are only limited longitudinal analyses of cross-season match play physical performance in professional players.

Longitudinal data from the Italian Serie-A (Mohr et al., 2003; Rampinini et al., 2007) indicate increases in match play TD, HSR, and very high-intensity running distances at the end of the season, which were attributed to low end-of-season game densities. Conversely, recent data from the German Bundesliga indicate cross season reductions in TD but increases in HSR toward the end of the season (Chmura et al., 2019). However, the competitive demands (total number of games, game density, and number of two-game weeks) in the Italian Serie-A and German Bundesliga are *very low* relative to the EC (Springham et al., 2019), and thus, these findings might not be generalizable across leagues. Indeed, league-specific longitudinal match play physical performance data are warranted for the EC.

Only one study has investigated cross-season match play physical performance in an EC cohort (Morgans et al., 2014a). This investigation reported a peak in average TD halfway through the season, but no other longitudinal changes. However, this investigation used home fixture data only, which can exert a confounding effect on physical performance (Lago-Penas, 2012; Carling, 2013). To date, no longitudinal investigations have statistically controlled for situational or contextual variables (i.e., match location, match outcome, quality of opposition, fixture density, and match goal deficit) that can exert an effect on match play physical performance (Lago-Penas, 2012; Carling, 2013)—this, despite such recommendations in the scientific literature (Carling, 2013).

Training and match load are also known to vary between playing positions (Dellal et al., 2011; Bradley et al., 2013; Kelly et al., 2019). For example, wide defenders (WD), central midfielders (CM), and wide midfielders (WM) have substantially greater TD, HSR, and SD demands during EC match play than central defenders (CD) and forwards (F) (Bradley et al., 2013). Accordingly, since prior load is known to relate to match play physical performance in professional players (Springham et al., 2020), cross-season match play physical performance changes might also vary between positions. However, no empirical data are available to describe position-specific cross-season changes in match play physical performance. Accordingly, the aims of this investigation were to report team average and positional changes to match play physical performance across an EC season while statistically controlling for situational and contextual variables.

## Materials and Methods

### Study Design

Match play physical performance was recorded across a complete 51-game, 40-week competitive season in 18 senior professional male outfield players (age = 23.3 ± 7.4 years; height = 180.2 ± 6.0 cm, body mass = 73.3 ± 6.3 kg) from one EC team. Of these players, three were central defenders (CD), four were wide defenders (WD), four were central midfielders (CM), four were wide midfielders (WM), and three were forwards (F). The season was divided into equal (10-week) quarters (Q) to facilitate longitudinal analysis: Q1: games 1–13, Q2: games 14–27, Q3: games 28–39, and Q4: games 40–51.

All competitive home and away games were filmed by fixed high-resolution, wide-angled cameras (Panasonic HC-V 100, Osaka, Japan), and a semiautomated computerized tracking system (InStat Fitness System, Moscow, Russia) was used to measure match play physical performance indices. The reliability [coefficient of variation (%)] of this system for the measurement of distance and instantaneous speed during linear and multidirectional running activities across a range of running velocities at different pitch locations in stadia is <1% (Alexeev et al., 2014).

Only data from players who played whole games were included in the analysis. Consistent with previous research literature (Morgans et al., 2014a), team average performance was calculated as the mean performance of all outfield players, per game. Positional average performance was calculated as the mean performance of players, grouped by playing position, per game. Data were omitted from six games in which a player was sent off for the sample or opposing team, from three games owing to technical error, and from one game in which extra time was played. Therefore, in total, data from 41 competitive games: 37 league, 2 domestic cup, and 2 league play-off games, were included in the analysis, equating to 368 player-match observations. For context, Q1 included 10 league and 1 domestic cup games; Q2 included 11 league games; Q3 included 8 league and 1 domestic cup games, and Q4 included 8 league and 2 domestic league playoff games. The number of whole games played by the 18 outfield players across the sample period is presented in Table 1. An ethics declaration was approved for this investigation by the Edith Cowan University (AU), Office of Research and Innovation.

**Table 1:** Average number of whole games played by the 18 outfield players across the sample period, arranged by playing position group.

**Playing position group**	***n***	**Number of whole games (Mean ± SD)**
Central defenders	3	31.3 (7.4)
Wide defenders	4	33.3 (16.7)
Central midfielders	4	31.0 (15.6)
Wide midfielders	4	25.3 (12.5)
Forwards	3	27.7 (22.4)

### Physical Performance Indices

Match play TD, work rate (WR), HSR, number of high-speed runs (NHSR), SD, and number of sprints (NS) were calculated for all players following games. Definitions for these are provided in Table 2.

**Table 2:** Definitions of match play running activities.

**Activity**	**Definition**
TD	Total distance completed per game (m)
WR	Average work rate per game (m / min)
HSR	Total distance completed between 5.5 and 7 m/s per game (m)
NHSR	Total number of running efforts between 5.5 and 7 m/s per game
SD	Total distance completed > 7 m/s per game (m)
NS	Total number of running efforts completed > 7 m/s per game (m)

### Situational and Contextual Variables

Match location (home or away), match outcome (win, draw, or loss), fixture density (number of days between games), and match goal deficit (positive value for a win, negative value for a loss) were recorded for each game. Post-season, league teams were assigned to high (top third, 1–8), intermediate (middle third, 9–16), or low (bottom third, 17–24) groups based on league position to determine quality of opposition.

### Statistical Analysis

All estimations were made using the *lme4* package (Bates et al., 2018) with *R* (version 3.5.1, R Foundation for Statistical Computing, Vienna, Austria). A linear mixed-effects model was used to model the effect of season quarter, playing position, and their interactions, upon each of the physical performance indices while adjusting for situational and contextual variables as covariates (additional fixed effects). The random effects were player identity (differences between players' mean output), player identity × season quarter (variability in the effect of season quarter across players), and the residual. The alpha level was set at *P* < 0.05. Data are presented as means and 95% confidence intervals (CI), alongside Cohen's *d* effect sizes (Hopkins et al., 2009). Thresholds for ES were: < 0.2 = *trivial*; 0.2–< 0.6 = *small*; 0.6–< 1.2 = *moderate*; 1.2–< 2 = *large*; ≥2 = *very large*. The *lmerTest* package (Kuznetsova et al., 2018) was used to conduct Bonferroni-adjusted pairwise comparisons for the main effect of playing position and phase of season, and their interactions.

## Results

### Team Season Average Match Play Physical Performance

Descriptive statistics for team season average match play physical performance are presented in Table 3.

**Table 3:** Descriptive statistics for team average match play physical performance indices by season quarter: TD, total distance; WR, work rate; HSR, high speed running; NHSR, number of high-speed runs; SD, sprint distance; NS, number of sprints.

	**Quarter1**		**Quarter 2**		**Quarter 3**		**Quarter 4**		**Season average**	
	**Mean (± SD)**	**CI**	**Mean (± SD)**	**CI**	**Mean (± SD)**	**CI**	**Mean (± SD)**	**CI**	**Mean (± SD)**	**CI**
TD	10,923 (748)	10,675–11,170	10,537 (818)	10,300–10,774	10,430 (824)	10,184–10,676	10,388 (931)	10,153–10,622	10,569 (830)	9,755–11,383
WR	113 (8.18)	111–116	111 (9.20)	108–113	108 (8.70)	105–110	107 (9.69)	104–109	109.75 (8.94)	101–119
HSR	969 (347)	836–1,102	827 (321)	697–957	873 (258)	740–1,005	929 (310)	799–1,058	900 (309)	597–1,202
NHSR	81.2 (28.9)	71.9–90.6	65.8 (21.2)	56.8–74.9	66.4 (21.3)	57.0–75.7	73.0 (19.5)	64.1–81.9	71.6 (22.7)	49.3–93.8
SD	196 (80.6)	168–224	196 (81.9)	170–223	158 (97.8)	130–186	132 (76.7)	106–158	171 (84.3)	88–253
NS	18.73 (8.2)	16.21–21.3	16.49 (6.7)	14.09–18.9	9.92 (6.4)	7.42–12.4	8.82 (5.3)	6.44–11.2	13.49 (6.7)	6.97–20.01

*Data are presented as mean ± SD and 95% CI*.

### Positional Season Average Match Play Physical Performance

Descriptive statistics for season average match play physical performance by playing position are presented in Table 4.

**Table 4:** Descriptive statistics for season average match play physical performance indices by playing position: TD, total distance; WR, work rate; HSR, high speed running; NHSR, number of high-speed runs; SD, sprint distance; NS, number of sprints.

	**Central defenders**		**Wide defenders**		**Central midfielders**		**Wide midfielders**		**Forwards**	
	**Mean (± SD)**	**CI**	**Mean (± SD)**	**CI**	**Mean (± SD)**	**CI**	**Mean (± SD)**	**CI**	**Mean (± SD)**	**CI**
TD	10,313 (576)	9,935–10,691	10,869 (632)	10,401–11,337	11,281 (673.5)	10,947–11,614	11,307 (704)	10,923–11,692	9,078 (966)	8,375–9,780
WR	106.8 (6.79)	102.8–111	112.8 (7.0)	107.8–118	116.9 (7.75)	113.3–120	117.6 (8.23)	113.5–122	93.6 (12.35)	86.1–101
HSR	758 (209)	534–984	970 (223)	694–1,247	1,054 (269)	858–1,250	1,200 (329)	973–1,426	513 (220)	111–914
NHSR	65.8 (20.3)	50.9–80.6	74.1 (20.3)	55.8–92.5	82.0 (20.1)	68.9–95.1	83.0 (26.5)	68.0–98.1	53.0 (19.1)	25.8–80.2
SD	106 (46.9)	68.3–144	188 (76.9)	140.8–236	160 (88.9)	126.0–194	232 (84.8)	192.8–271	167 (99.1)	92.8–241
NS	8.64 (4.8)	4.85–12.4	14.84 (7.9)	10.15–19.5	12.93 (8.17)	9.58–16.3	17.05 (7.73)	13.19–20.9	13.99 (8.41)	6.91–21.1

*Data are presented as mean ± SD and 95% CI*.

**Figure 1:**
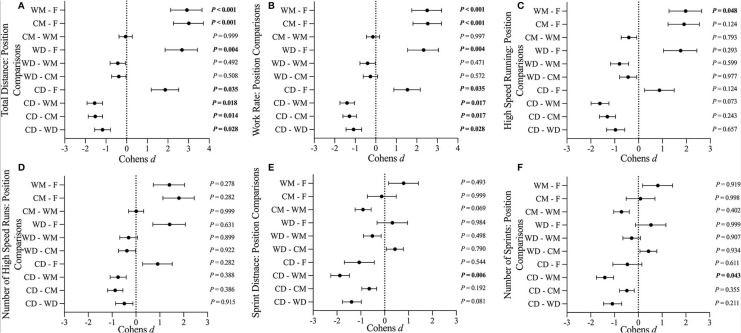
**(A–F)** Standardized differences in season average match play physical performance measures between playing positions: CD, central defenders; WD, wide defenders; CM, central midfielders; WM, wide midfielders; F, forwards. Data are presented as the standardized Cohens *d* effect size ± 95% CI.

Differences in season average match play physical performance measures between playing positions are presented in Figure 1. WD completed greater TD than CD (*P* = 0.028, ES = *Moderate*) and F (*P* = 0.004, ES = *Very Large*) (Figure 1A). CM completed greater TD than CD (*P* = 0.014, ES = *Large*) and F (*P* < 0.001, ES = *Very Large*) (Figure 1A). WM completed greater TD than CD (*P* = 0.018, ES = *Very Large*) and F (*P* < 0.001, ES = *Very Large*) (Figure 1A). CD completed greater TD than F (*P* = 0.035, ES = *Large*) (Figure 1A). WD completed greater WR than CD (*P* = 0.028, ES = *Moderate*) and F (*P* = 0.004, ES = *Very Large*) (Figure 1B). CM completed greater WR than CD (*P* = 0.017, ES = *Large*) and F (*P* < 0.001, ES = *Very Large*) (Figure 1B). WM completed greater WR than CD (*P* = 0.017, ES = *Very Large*) and F (*P* < 0.001, ES = *Very Large*) (Figure 1B). CD completed greater WR than F (*P* = 0.035, ES = *Large*) (Figure 1B). WM completed greater HSR than F (*P* = 0.048, ES = *Large*) (Figure 1C). WM completed greater SD than CD (*P* = 0.006, ES = *Large*) (Figure 1E). WM completed greater NS than CD (*P* = 0.043, ES = *Large*) (Figure 1F).

### Team Average Match Play Physical Performance by Season Quarter

Descriptive statistics for team average physical performance by season quarter are presented in Table 3. Differences in team average match play physical performance variables by season quarter are presented in Table 5.

**Table 5:** Team average and positional match physical performance changes: season quarter comparisons.

	**Q1 vs. Q2**			**Q1 vs. Q3**			**Q1 vs. Q4**			**Q2 vs. Q3**			**Q2 vs. Q4**			**Q3 vs. Q4**			**Summary**
	**Effect**	***P***	**ES**	**Effect**	***P***	**ES**	**Effect**	***P***	**ES**	**Effect**	***P***	**ES**	**Effect**	***P***	**ES**	**Effect**	***P***	**ES**	
**Total distance**																			
*CD*	Q1 > Q2	> 0.05	Small	Q1 > Q3	> 0.05	Moderate	Q1 > Q4	> 0.05	Moderate	Q2 > Q3	> 0.05	Small	Q2 > Q4	> 0.05	Small	Q3 < Q4	> 0.05	Trivial	
WD	Q1 > Q2	> 0.05	Small	Q1 > Q3	> 0.05	Moderate	Q1 > Q4	> 0.05	Small	Q2 > Q3	> 0.05	Trivial	Q2 < Q4	> 0.05	Trivial	Q3 < Q4	> 0.05	Small	
CM	Q1 > Q2	> 0.05	Moderate	Q1 > Q3	> 0.05	Large	Q1 > Q4	> 0.05	Moderate	Q2 > Q3	> 0.05	Small	Q2 > Q4	> 0.05	Trivial	Q3 > Q4	> 0.05	Trivial	
WM	Q1 > Q2	> 0.05	Small	Q1 > Q3	> 0.05	Small	Q1 > Q4	> 0.05	Small	Q2 < Q3	> 0.05	Trivial	Q2 > Q4	> 0.05	Trivial	Q3 > Q4	> 0.05	Small	
F	Q1 > Q2	> 0.05	Large	Q1 > Q3	> 0.05	Large	Q1 > Q4	> 0.05	Large	Q2 < Q3	> 0.05	Small	Q2 < Q4	> 0.05	Trivial	Q3 > Q4	> 0.05	Small	
TA	Q1 > Q2	**0.002**	Moderate	Q1 > Q3	**< 0.001**	Moderate	Q1 > Q4	**< 0.001**	Moderate	Q2 > Q3	> 0.05	Trivial	Q2 > Q4	> 0.05	Trivial	Q3 > Q4	> 0.05	Trivial	Q1 > Q2, Q3, Q4
**Work rate**																			
CD	Q1 > Q2	> 0.05	Trivial	Q1 > Q3	> 0.05	Moderate	Q1 > Q4	> 0.05	Large	Q2 > Q3	> 0.05	Moderate	Q2 > Q4	> 0.05	Moderate	Q3 > Q4	> 0.05	Trivial	
WD	Q1 > Q2	> 0.05	Small	Q1 > Q3	> 0.05	Moderate	Q1 > Q4	> 0.05	Moderate	Q2 > Q3	> 0.05	Small	Q2 > Q4	> 0.05	Small	Q3 < Q4	> 0.05	Trivial	
CM	Q1 > Q2	> 0.05	Moderate	Q1 > Q3	> 0.05	Moderate	Q1 > Q4	**0.020**	Large	Q2 > Q3	> 0.05	Small	Q2 > Q4	> 0.05	Moderate	Q3 > Q4	> 0.05	Small	Q1 > Q4
WM	Q1 > Q2	> 0.05	Small	Q1 > Q3	> 0.05	Small	Q1 > Q4	> 0.05	Moderate	Q2 > Q3	> 0.05	Trivial	Q2 > Q4	> 0.05	Small	Q3 > Q4	> 0.05	Small	
F	Q1 > Q2	> 0.05	Moderate	Q1 > Q3	> 0.05	Large	Q1 > Q4	> 0.05	Large	Q2 < Q3	> 0.05	Small	Q2 < Q4	> 0.05	Trivial	Q3 > Q4	> 0.05	Small	
TA	Q1 > Q2	> 0.05	Moderate	Q1 > Q3	**< 0.001**	Moderate	Q1 > Q4	**< 0.001**	Large	Q2 > Q3	> 0.05	Trivial	Q2 > Q4	**0.003**	Small	Q3 > Q4	> 0.05	Trivial	Q1 > Q3, Q4; Q2 > Q4
**High-speed running distance**																			
CD	Q1 > Q2	> 0.05	Moderate	Q1 > Q3	> 0.05	Small	Q1 > Q4	> 0.05	Trivial	Q2 < Q3	> 0.05	Small	Q2 < Q4	> 0.05	Moderate	Q3 < Q4	> 0.05	Moderate	
WD	Q1 > Q2	> 0.05	Small	Q1 > Q3	> 0.05	Trivial	Q1 < Q4	> 0.05	Trivial	Q2 < Q3	> 0.05	Small	Q2 < Q4	> 0.05	Small	Q3 < Q4	> 0.05	Trivial	
CM	Q1 > Q2	> 0.05	Small	Q1 > Q3	> 0.05	Small	Q1 > Q4	> 0.05	Trivial	Q2 < Q3	> 0.05	Small	Q2 < Q4	> 0.05	Small	Q3 < Q4	> 0.05	Trivial	
WM	Q1 > Q2	> 0.05	Small	Q1 > Q3	> 0.05	Small	Q1 > Q4	> 0.05	Small	Q2 < Q3	> 0.05	Trivial	Q2 < Q4	> 0.05	Small	Q3 < Q4	> 0.05	Small	
F	Q1 > Q2	> 0.05	V Large	Q1 > Q3	> 0.05	V Large	Q1 > Q4	> 0.05	V Large	Q2 > Q3	> 0.05	Small	Q2 < Q4	> 0.05	Large	Q3 < Q4	> 0.05	V Large	
TA	Q1 > Q2	**0.006**	Moderate	Q1 > Q3	> 0.05	Moderate	Q1 > Q4	> 0.05	Small	Q2 < Q3	> 0.05	Trivial	Q2 < Q4	> 0.05	Small	Q3 < Q4	> 0.05	Small	Q1 > Q2
**Number of high-speed runs**																			
CD	Q1 > Q2	> 0.05	Moderate	Q1 > Q3	> 0.05	Moderate	Q1 > Q4	> 0.05	Small	Q2 > Q3	> 0.05	Trivial	Q2 < Q4	> 0.05	Small	Q3 < Q4	> 0.05	Moderate	
WD	Q1 > Q2	> 0.05	Small	Q1 > Q3	> 0.05	Small	Q1 > Q4	> 0.05	Trivial	Q2 > Q3	> 0.05	Trivial	Q2 < Q4	> 0.05	Small	Q3 < Q4	> 0.05	Small	
CM	Q1 > Q2	> 0.05	Moderate	Q1 > Q3	> 0.05	Small	Q1 > Q4	**< 0.05**	Small	Q2 < Q3	> 0.05	Small	Q2 < Q4	> 0.05	Small	Q3 < Q4	> 0.05	Trivial	
WM	Q1 > Q2	> 0.05	Moderate	Q1 > Q3	> 0.05	Small	Q1 > Q4	> 0.05	Small	Q2 < Q3	> 0.05	Trivial	Q2 < Q4	> 0.05	Moderate	Q3 < Q4	> 0.05	Small	
F	Q1 > Q2	> 0.05	V Large	Q1 > Q3	> 0.05	V Large	Q1 > Q4	> 0.05	V Large	Q2 > Q3	> 0.05	Large	Q2 < Q4	> 0.05	Large	Q3 < Q4	> 0.05	V Large	
TA	Q1 > Q2	**< 0.001**	Moderate	Q1 > Q3	**0.002**	Moderate	Q1 > Q4	> 0.05	Moderate	Q2 > Q3	> 0.05	Trivial	Q2 < Q4	> 0.05	Small	Q3 < Q4	> 0.05	Moderate	Q1 > Q2, Q3
**Sprint distance**																			
CD	Q1 < Q2	> 0.05	Trivial	Q1 > Q3	> 0.05	Moderate	Q1 > Q4	> 0.05	Moderate	Q2 > Q3	> 0.05	Moderate	Q2 > Q4	> 0.05	Large	Q3 > Q4	> 0.05	Small	
WD	Q1 > Q2	> 0.05	Small	Q1 > Q3	> 0.05	Small	Q1 > Q4	> 0.05	Large	Q2 < Q3	> 0.05	Trivial	Q2 > Q4	> 0.05	Large	Q3 < Q4	> 0.05	Moderate	
CM	Q1 < Q2	> 0.05	Trivial	Q1 > Q3	> 0.05	Moderate	Q1 > Q4	**< 0.05**	Moderate	Q2 > Q3	> 0.05	Moderate	Q2 > Q4	> 0.05	Moderate	Q3 > Q4	> 0.05	Trivial	
WM	Q1 < Q2	> 0.05	Small	Q1 > Q3	> 0.05	Small	Q1 > Q4	> 0.05	Moderate	Q2 > Q3	> 0.05	Small	Q2 > Q4	> 0.05	Moderate	Q3 > Q4	> 0.05	Small	
F	Q1 > Q2	> 0.05	Moderate	Q1 > Q3	> 0.05	Moderate	Q1 > Q4	> 0.05	Moderate	Q2 < Q3	> 0.05	Small	Q2 < Q4	> 0.05	Small	Q3 > Q4	> 0.05	Small	
TA	Q1 > Q2	> 0.05	Small	Q1 > Q3	> 0.05	Small	Q1 > Q4	**< 0.001**	Moderate	Q2 > Q3	**0.035**	Trivial	Q2 > Q4	**< 0.001**	Moderate	Q3 > Q4	> 0.05	Small	Q1 > Q4, Q2 > Q3, Q4
**Number of sprints**																			
CD	Q1 > Q2	> 0.05	Small	Q1 > Q3	> 0.05	Large	Q1 > Q4	**0.013**	Large	Q2 > Q3	> 0.05	Large	Q2 > Q4	> 0.05	V Large	Q3 > Q4	> 0.05	Trivial	Q1 > Q4
WD	Q1 > Q2	> 0.05	Moderate	Q1 > Q3	**0.046**	Large	Q1 > Q4	**< 0.001**	V Large	Q2 > Q3	> 0.05	Moderate	Q2 > Q4	**0.043**	V Large	Q3 > Q4	> 0.05	Moderate	Q1 > Q3, Q4; Q2 > Q4
CM	Q1 > Q2	> 0.05	Trivial	Q1 > Q3	**0.002**	Moderate	Q1 > Q4	**0.004**	Large	Q2 > Q3	**0.002**	Large	Q2 > Q4	**0.004**	Large	Q3 < Q4	> 0.05	Trivial	Q1 > Q3, Q4; Q2 > Q3, Q4
WM	Q1 < Q2	> 0.05	Trivial	Q1 > Q3	> 0.05	Moderate	Q1 > Q4	**0.012**	Moderate	Q2 > Q3	> 0.05	Moderate	Q2 > Q4	**0.007**	Large	Q3 > Q4	> 0.05	Small	Q1 > Q4; Q2 > Q4
F	Q1 > Q2	> 0.05	V Large	Q1 > Q3	> 0.05	V Large	Q1 > Q4	> 0.05	V Large	Q2 < Q3	> 0.05	Small	Q2 > Q4	> 0.05	Small	Q3 > Q4	> 0.05	Moderate	
TA	Q1 > Q2	> 0.05	Moderate	Q1 > Q3	**< 0.001**	Large	Q1 > Q4	**< 0.001**	Large	Q2 > Q3	**< 0.001**	Moderate	Q2 > Q4	**< 0.001**	Large	Q3 > Q4	> 0.05	Trivial	Q1 > Q3, Q4; Q2 > Q3, Q4

*Q1, quarter 1; Q2, quarter 2; Q3, quarter 3; Q4, quarter 4. CD, central defenders; WD, wide defenders; CM, central midfielders; WM, wide midfielders; F, forwards; TA, team average. Q1, quarter 1; Q2, quarter 2; Q3, quarter 3; Q4, quarter 4*.

Reductions in team average TD were observed between Q1 and Q2 (*P* = 0.002, ES = *Moderate*), Q1 and Q3 (*P* < 0.001, ES = *Moderate*), and Q1 and Q4 (*P* < 0.001, ES = *Moderate*) (Table 5). Reductions in team average WR were observed between Q1 and Q3 (*P* < 0.001, ES = *Moderate*), Q1 and Q4 (*P* < 0.001, ES = *Large*), and Q2 and Q4 (*P* = 0.003, ES = *Small*) (Table 5). Reductions in team average HSR were observed between Q1 and Q2 (*P* = 0.006, *ES* = *Moderate*) (Table 5). Team average NHSR were reduced between Q1 and Q2 (*P* < 0.001, ES *Moderate*) and Q1 and Q3 (*P* = 0.002, ES = *Moderate)* (Table 5). Reductions in team average SD were observed between Q1 and Q4 (*P* < 0.001, ES = *Moderate*), Q2 and Q3 (*P* < 0.035, ES = *Small*), and Q2 and Q4 (*P* < 0.001, ES = *Moderate*) (Table 5). Team average NS were reduced between Q1 and Q3 (*P* < 0.001, ES = *Moderate*), Q1 and Q4 (*P* < 0.001, ES = *Large*), Q2 and Q3 (*P* < 0.001, ES = *Trivial*), and Q2 and Q4 (*P* < 0.001, ES = *Moderate*) (Table 5).

### Positional Match Play Physical Performance by Season Quarter

**Figure 2:**
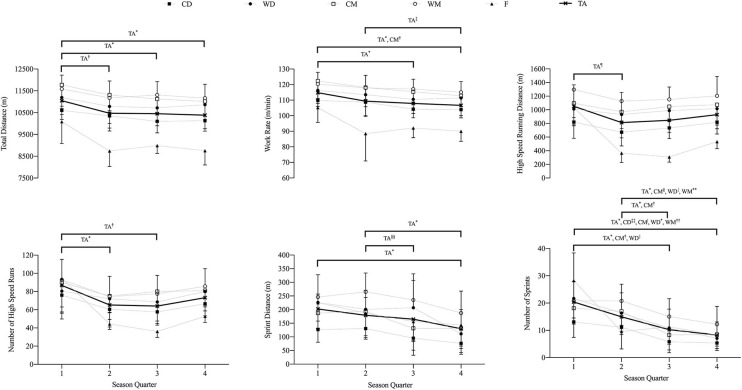
Team average and positional physical performance by season quarter: CD, central defenders; WD, wide defenders; CM, central midfielders; WM, wide midfielders; F, forwards; TA, team average. Data are presented as mean ± SD. Horizontal lines and symbols denote differences within groups: *P = *0.001;* ^†^*P* = *0.002;* ^‡^*P* = *0.003;* ^§^*P* = *0.004;* ^|^*P* = *0.005;* ^¶^*P* = *0.006;* **P = *0.007; P* = *0.012;* ^‡‡^*P* = *0.014;* ^§§^*P* = *0.035;* ^||^*P* = *0.044*.

Match play physical performance data by playing position and season quarter are presented in Figure 2. Magnitudes of cross-season match play physical performance changes are presented in Table 5. WR was reduced in CM between Q1 and Q4 (*P* = 0.020, ES = *Large*) (Table 5). Reductions in NS were observed between Q1 and Q3 in WD (*P* = 0.046, ES = *Large*) and CM (*P* = 0.002, ES = *Moderate*) (Table 5). Reductions in NS between Q1 and Q4 were observed in CD (*P* = 0.013, ES = *Large*), WD (*P* < 0.001, ES = *Very Large*), CM (*P* = 0.004, ES = *Large*), and WM (*P* = 0.012, ES = *Moderate*) (Table 5). Reductions in NS were observed between Q2 and Q3 in CM (*P* = 0.002, ES = *Large*) and between Q2 and Q4 in WD (*P* = 0.043, ES = *Very Large*), CM (*P* = 0.004, ES =*Large*), and WM (*P* = 0.007, ES = *Large*) (Table 5).

## Discussion

The first aim of this investigation was to examine team average match play physical performance across an EC season. Our result is a decrease in all physical performance indices across the season. The second aim was to report positional changes in match play physical performance. We observed decreases in match play physical performance indices across the season in all positions. To our knowledge, this is the first investigation to report longitudinal decreases in match play physical performance across a professional football season at team average and positional levels while controlling for situational and contextual variables.

Importantly, the physical demands of match play herein are consistent with previously published data from the EC (Bradley et al., 2013; Di Salvo et al., 2013). For example, season team average TD, HSR, and SD herein were 10,569 ± 830 m, 900 ± 309 m, and 171 ± 84 m, respectively (Table 3), which are similar to the data reported by Bradley et al. (2013) (11,429 ± 816 m, 803 ± 227 m, and 308 ± 139 m) and Di Salvo et al. (2013) (11,102 ± 916 m, 750 ± 222 m, and 273 ± 125 m). Accordingly, match load demands in the current investigation are typical for the EC.

The major finding in this investigation is a cross-season decrease in team average match play physical performance, spanning all measures (Figure 2). This finding is contrary to existing data from the EC (Morgans et al., 2014a) and other European leagues (Mohr et al., 2003; Rampinini et al., 2007; Chmura et al., 2019). Of note, the team head coach, coaching staff, and sport science and medical staff remained constant across the sample period. Moreover, team tactics and the tactical and physical preparation methods employed by the team were constant across the sample period. In addition, our statistical analysis controlled for situational and contextual variables known to affect match play physical performance (Lago-Penas, 2012; Carling, 2013). As such, it is likely that the decreases in performance observed herein are explained by factors internal to the playing cohort.

Relative to the other major European football leagues, EC teams play the highest number of total games, have the greatest game density, and have the largest number of two-game weeks per season (Springham et al., 2019). Consequently, regularly selected EC players often have a limited recovery period between games (~72 h) and are likely to play a substantial number of games in an underrecovered state. This, in turn, might increase the risk of fatigue, maladaptive training, and non-functional overreaching (Meeusen et al., 2013; Schwellnus et al., 2016; Soligard et al., 2016). This cascade is known to cause changes to biological, neurochemical, and hormonal regulation mechanisms and compromises physical performance potential (Meeusen et al., 2013; Schwellnus et al., 2016; Soligard et al., 2016). Accordingly, the cross-season decreases in match play physical performance observed herein might be explained by longitudinal fatigue.

Only one other investigation has reported the seasonal kinetics of match play physical performance in the EC. Morgans et al. (2014a) recorded match play physical performance in home games across a single season and reported a peak in team TD halfway through the season, but no other longitudinal changes to physical performance indices. However, match location can exert a confounding effect on match play physical performance (Lago-Penas, 2012; Carling, 2013). Indeed, players are more likely to complete greater total match distance when playing at home compared to when playing away (Lago-Penas, 2012), owing to the combined effects of crowd, travel, familiarity, referee bias, territoriality, specific tactics, and psychological factors (Lago-Penas, 2012). Accordingly, the use of home game data alone might not be suitable for determining cross-season match-related fatigue. Indeed, this might explain the discrepancies between our findings and those reported previously (Morgans et al., 2014a).

As well as match location, our statistical analysis controlled for match outcome, goal deficit, quality of opposition, and fixture density, in line with previous recommendations (Carling, 2013). Match outcome and goal deficit can influence player and team match strategies (Lago and Martin, 2007; Taylor et al., 2008; Lago-Penas, 2012) whereby players are more likely to adopt defensive characteristics and complete less high-intensity activity when winning by larger margins as opposed to losing by smaller margins or when drawing (Lago and Martin, 2007; Taylor et al., 2008; Lago-Penas, 2012). Data also indicate that players complete greater HSR when playing against higher-quality opposition (Rampinini et al., 2007) and, conversely, lower TD when competing against lower-quality opposition (Lago-Penas, 2012). It is also proposed that players can self-regulate physical activity during congested periods to moderate fatigue (Rampinini et al., 2007; Castellano et al., 2011). Accordingly, the inclusion of these covariates into our statistical analysis might help further explain the discrepancies between our observations and those reported previously (Mohr et al., 2003; Rampinini et al., 2007; Morgans et al., 2014a) and support the notion of a cross-season fatiguing effect herein.

Team average data indicate *moderate*-to-*large* cross-season changes for sprint performance indices (SD and NS) (Table 5) and *trivial*-to-*moderate* changes for TD, HSR, and NHSR (Table 5). This suggests that players are able to maintain low- to high-speed performance (< 7 m/s) but less able to maintain sprinting performance (> 7 m/s) cross-season. These findings are consistent with other recent scientific research, reporting end-of-season reductions in match play sprint performance in elite-level professional football players (Chmura et al., 2019). Football match play and training are characterized by repetitive, high-intensity, moderate-to-high force stretch-shortening cycle activities, including acceleration, deceleration, change in direction, and sprinting (Akenhead et al., 2013). These are proposed to be the dominant causal activities of low-frequency or “neuromuscular” fatigue in athletes (Fowles, 2006), which reduces the rate of force development during maximal efforts and power output during sustained dynamic actions (Fowles, 2006). Therefore, it follows that neuromuscular fatigue might manifest as a reduction in match play sprinting activity in football. Cross-season decreases in neuromuscular performance have previously been observed during an Australian Rules Football (AFL) season (Cormack et al., 2008). Accordingly, neuromuscular fatigue is a viable explanatory candidate for the decreases observed in SD and NS herein. These findings are of practical importance to teams, owing to the decisive role that player sprint performance has during football match play (Bradley et al., 2009; Di Salvo et al., 2010) and in contributing to match outcome (Andrzejewski et al., 2018).

Previous scientific literature have proposed that players can utilize conscious or subconscious pacing strategies to maintain high-speed output across games by reducing concurrent low-speed output (Folgado et al., 2015; Jones et al., 2018). For example, a reduction in “low” -speed distance (< 4 m/s) but maintenance of “high” -speed distance (> 4 m/s) has been reported during isolated periods of high fixture density (Folgado et al., 2015). Recent longitudinal data indicate that players might also employ pacing strategies to manage cross-season match play physical performance. For example, data from the German Bundesliga indicate cross-season reductions in match play low-speed performance, but concurrent cross-season increases high-speed performance (Chmura et al., 2019). Interestingly, consistent with the current investigation, this study also reported substantial reductions in sprint performance at the end of the competitive season (Chmura et al., 2019). Collectively, findings from the current and previous (Chmura et al., 2019) investigations suggest that offsetting a longitudinal decline in sprinting activity (> 7 m/s) might not always be possible in the context of complete competitive seasons.

Our results indicate that “high-load” positions can experience greater cross-season decreases in match play physical performance than “low-load” positions. For example, consistent with previous literature (Bradley et al., 2013), we observed greater season average match loads in WD, CM, and WM than in CD and F (Table 4, Figure 1). Within positions, our results indicate greater cross-season reductions for WR and NS in “high-load” (WD, CM and WM) than “low-load” playing positions (CD and F) (Figure 2). This suggests a relationship between the rate of match load accumulation and the rate of cross-season deterioration in match play physical performance. Recent data indicate that very high absolute sprint workloads compromise match play sprint performance in elite-level professional football players, likely related to fatigue (Springham et al., 2020). Accordingly, that cross-season match play sprint performance deteriorated most in positions with the greatest sprint demands (Figure 1) is unsurprising. This finding highlights a particular vulnerability for cross-season match play sprint performance deterioration in high-load positions (WD, CM, and WM).

The authors acknowledge that the use of global, as opposed to individualized, running speed thresholds is a major limitation of this investigation. Other limitations include the absence of training load, fatigue, and fitness profiling data. The authors acknowledge that other players and teams might respond differently owing to interteam factors.

## Practical Applications

We observed *very large* cross-season reductions in match play physical performance. Accordingly, physical periodization and squad rotation strategies should be considered to regulate workload, manage fatigue, and mitigate longitudinal risks to match play physical performance. This is particularly important during periods of high game density and two-game weeks, when less time is available for players to recover between games. The authors acknowledge that the capacity to rotate players might be limited by factors including the quality of opposition, player availability, player quality, and coaching philosophy.

We observed *very large* cross-season reductions in sprint performance indices (SD, NS). Since neuromuscular fatigue is a likely cause, we propose that practitioners should monitor and regulate high-speed and high-intensity workload (acceleration, deceleration, sprinting, and HSR activity) and regularly monitor neuromuscular fatigue status in professional players. This might facilitate improved player management decisions and mitigate the risk of longitudinal decreases in player sprint performance.

Finally, practitioners should develop repeated sprint capacity in players. This might mitigate cross-season reductions in match play sprint performance.

## Conclusion

Team average match play physical performance decreased across an EC season. The most notable decreases in performance were observed in sprint performance indices (SD, NS) for which the greatest reductions were observed in WD, CM, and WM.

## Data Availability Statement

The datasets presented in this article are not readily available because of ethical and privacy restraints relating to the sample team. Requests to access the datasets should be directed to Matthew Springham, matt.springham@stmarys.ac.uk.

## Ethics Statement

The studies involving human participants were reviewed and approved by Edith Cowan University Office for Research and Innovation. The patients/participants provided their written informed consent to participate in this study.

## Author Contributions

All authors contributed to the conception and design of the work. MS completed the acquisition, analysis, and interpretation of the data for the work. SW completed the analysis of the data. All authors drafted the work or revised it critically for important intellectual content, approved the final version of the manuscript, and agreed to be accountable for all aspects of the work ensuring accuracy and integrity.

## Conflict of Interest

The authors declare that the research was conducted in the absence of any commercial or financial relationships that could be construed as a potential conflict of interest.
